# Assessing the Acceptability and Feasibility of a Web-Based Screening for Psychoactive Substance Users Among a French Sample of University Students and Workers: Mixed Methods Prospective Study

**DOI:** 10.2196/15519

**Published:** 2021-10-01

**Authors:** Emmanuelle Anthoine, Julie Caillon, Xavier Deparis, Michel Blanche, Maxime Lebeaupin, Marc-Antoine Brochard, Jean-Luc Venisse, Leïla Moret

**Affiliations:** 1 PHU11 Public Health Department Centre Hospitalier Universitaire (CHU) de Nantes Nantes France; 2 Addictions Department Centre Hospitalier Universitaire (CHU) de Nantes Nantes France; 3 Service de Santé au Travail de la Région Nantaise Nantes France; 4 Service de Santé des Étudiants (SUMPPS) University of Nantes Nantes France; 5 Pulsio Santé Stimulab Company Paris France; 6 UMR 1246 SPHERE (Methods in Patient-Centered Outcomes and Health Research) University of Nantes and Tours Nantes France

**Keywords:** feasibility study, preventive medicine, addiction, screening and brief intervention, web-based app

## Abstract

**Background:**

Early detection in the prevention of addictive behaviors remains a complex question in practice for most first-line health care workers (HCWs). Several prevention measures have successfully included a screening stage followed by a brief intervention in case of risk-related use or referral to an addiction center for problematic use. Whereas early detection is highly recommended by the World Health Organization, it is not usually performed in practice.

**Objective:**

The aim of this study was to assess the acceptability and feasibility of a web-based app, called Pulsio Santé, for health service users and first-line prevention HCW and to carry out an exhaustive process of early detection of psychoactive substance use behaviors.

**Methods:**

A mixed methods prospective study was conducted in 2 departments: HCWs from the regional occupational health department and from the university department of preventive medicine dedicated to students were invited to participate. Participants 18 years or older who had been seen in 2017 by a HCW from one of the departments were eligible. The study procedure comprised 5 phases: (1) inclusion of the participants after a face-to-face consultation with an HCW; (2) reception of a text message by participants on their smartphone or by email; (3) self-assessment by participants regarding their substance use with the Pulsio Santé app; (4) if participants agreed, transfer of the results to the HCW; and (5) if participants declined, a message to invite them to get in touch with their general practitioner should the assessment detect a risk. Several feasibility and acceptability criteria were assessed by an analysis of a focus group with the HCW that explored 4 themes (usefulness and advantages, problems and limitations, possible improvements, and finally, integration into routine practice).

**Results:**

A total of 1474 people were asked to participate, with 42 HCWs being involved. The percentage of people who agreed to receive a text message or an email, which was considered as the first level of acceptability, was 79.17% (1167/1474). The percentage of participants who clicked on the self-assessment link, considered as the second level of acceptability, was 60.24% (703/1167). The percentage of participants who completed their self-evaluation entirely, which was considered as the first level of feasibility, was 76.24% (536/703). The percentage of participants who shared the results of their evaluation with the HCWs, considered as the second level of feasibility, was 79.48% (426/536). The qualitative study showed that there were obstacles on the side of HCWs in carrying out the recommended interventions for people at risk based on their online screening, such as previous training or adaptations in accordance with specific populations.

**Conclusions:**

Quantitative results showed good acceptability and feasibility of the Pulsio Santé app by users and HCWs. There is a need for further studies more directly focused on the limitations highlighted by the qualitative results.

## Introduction

Today, use of psychoactive substances is a major problem everywhere in the world and is insufficiently dealt with and taken into account too late from the perspective of damage prevention and risk reduction [1-3]. Besides tobacco, other examples of psychoactive substances include alcohol, cannabis, cocaine, amphetamines, and hallucinogenic drugs. In France, for example, it is estimated that 14 million people are daily smokers, 3.4 million engage in risky alcohol consumption, and 1.5 million use cannabis regularly (according to data produced in 2019 by the French Monitoring Center for Drugs and Drug Addiction) [4]. Moreover, 75,000 tobacco-related deaths and 41,000 alcohol-related deaths occur each year. Psychoactive substance use opens the door to addictive behaviors, which can be divided into 2 types: risk-related use and problematic use [5].

Risk-related use is defined as use involving a particular risk according to the characteristics of its usage (quantity, frequency, and nature), the context in which it is used (eg, when driving a car or doing precision work on hazardous machines), and the presence of particular vulnerability factors (eg, young age, pregnancy or psychiatric pathology, treatment). Problematic use corresponds to harmful usage or the causing of detectable damage, whether somatic, psychological, socio-professional, familial, or that characterized by a syndrome of pathological dependence [6].

Among existing prevention strategies, certain prevention programs have successfully included a detection stage followed by a brief intervention stage in case of risk-related use. Miller’s team [7] defined a brief intervention in 6 points grouped under the acronym FRAMES *(*Feedback, Responsibility, Advise, Menu for Change, Empathy, and Enhancing Self-Efficacy). The brief intervention includes detailed feedback of the results of the evaluation and the risks incurred. It further includes the friendly assertion that the responsibility for change lies with the service user concerned, that he or she is in the best position to design and program this change, and that the reduction in consumption is an accessible objective within the means of the person's own resources [8]. Brief interventions are usually conducted in a one-on-one situation in primary care. They can be implemented anywhere on the intervention continuum and are based on a short, evidence-based, structured conversation consisting of personalized feedback and counseling adjusted to the patient's risk.

Although early detection has been highly recommended for 20 years by the World Health Organization (WHO) [9], primary health care professionals, specifically general practitioners, face difficulties in identifying the use of psychoactive substances, particularly alcohol and cannabis. Despite tobacco use often appearing in patients' records, there is little documentation of alcohol or cannabis use. Existing tracking questionnaires are little used in practice. Moreover, general practitioners currently face difficulties in the feasibility of additional systematic screening, and the implementation of a brief intervention would double the length of a consultation. In fact, general practitioners are not yet sufficiently assuming their role of prevention, which is often attributable to a lack of training [10,11].

It is thus reasonable to believe a web-based app could facilitate the work of primary health care professionals by helping them with early detection and brief interventions. Indeed, a web-based app could simplify the work of professionals by offering patients anonymous but standardized assessments of behaviors that are potentially risky for substance users and by providing tailored backup for health professionals in their interventions.

There is a growing body of literature exploring the use of apps to enhance the feasibility, reliability, and cultural acceptance of screening tests for possible overuse of psychoactive substances. Focusing mostly on a single substance (first and foremost alcohol), they have extended their scope of study to all other substances—medical drugs included—thanks to the WHO-developed Alcohol, Smoking and Substance Involvement Screening Test (ASSIST) [12,13]

Researchers also investigated how much these web-based apps can facilitate the realization of recommended brief interventions once a risk-prone usage has been identified—face-to-face with the patient or online [14-18]

The role of such apps in mediating the relationship between a service user and a health professional still needs to be evaluated, most notably when it comes to the level of autonomy and responsibility it provides to the service user (eg, giving service users the choice to share his or her results). The conditions enabling the realistic implementation of these apps in the real-life practice of professionals in the environments where they consult has also yet to be scrutinized to complement the latest publications [19].

The following study is therefore the first French study aimed at tackling these issues.

Pulsio Santé and Centre Hospitalier Universitaire de Nantes committed to an agreement to perform the study through a collaborative research project.

The main objective of this study was to assess the acceptability and feasibility of the Pulsio Santé web-based app by service users and first-line health professionals involved in prevention for exhaustive early detection of psychoactive substance use behaviors. The secondary objective was to assess whether the Pulsio Santé app could contribute to facilitating the recourse to brief interventions or referral to addiction centers if the results of the assessment pointed to the app’s usefulness.

## Methods

A mixed methods prospective study was conducted. It was carried out by a project team composed of senior and junior psychiatrists (JLV and JC, respectively), a senior public health physician (LM), a computer scientist (MAB), a project manager (ML), and a statistician (EA). The head of the Occupational Health Department (XD) and the head of the University Department of Preventive Medicine (MB) also contributed to the study.

### Characteristics of the Sample

#### Departments and Health Professionals

Two departments of health professionals volunteered to participate in the study: (1) The Occupational Health Department for the Nantes Region (Service de Santé au Travail de la Région Nantaise [SSTRN]), which is the biggest occupational health department in the county district and is responsible for managing the health of salaried workers in the region—indeed, the issue of risk-related and problematic psychoactive substance use has become a major concern in the business world [20]; (2) The Nantes City University Department of Preventive Medicine and Health Promotion (Service de Santé des Étudiants [SUMPPS]), one of the health departments within the university dedicated to students who are particularly concerned by this type of consumption due to the students’ age range [21,22]. In each department, all health professionals (physicians and nurses) were invited to participate as investigators in the study without altering their work schedules. Participating in the study as investigators was voluntary and unpaid.

#### Participants

Service users 18 years or older who were seen by a health professional either in the SSTRN or in the SUMPPS in the fourth quarter of 2017 were eligible. Participation in the study was voluntary and unpaid to prevent any monetary bias.

The following exclusion criteria were used to screen out unsuitable study participants: insufficient knowledge of the French language, no access or inability to use a web-based app, under 18 years old or under guardianship, ongoing treatment in a specialized addiction care unit, or disorder of the higher level cognitive functions or cognitive dysfunction (particularly in the area of psychoses) that would make the use of a web-based app difficult.

### Description of the Pulsio Santé Web-Based App

The Pulsio Santé web-based app can be accessed from either a laptop, tablet, or smartphone. Apart from ownership of one of these devices, it only requires internet access. The app does not have to be downloaded, and no account creation by the participant is necessary. For this ease of access, it offers completion of a whole screening test, provides one’s results, and enables understanding of the underlying risks without having to enter any identifying information. Only at the end of this process does it offer those participants who would like to discuss their results with a health professional the possibility of creating an account and requesting an appointment. All data are stored on health data–compliant servers located in France.

The Pulsio Santé study comprised 4 implementation stages: inclusion by health professionals, self-assessment by the users of their substance use, analysis of the results, and implementation of a tailored intervention if required. The app is based on the ASSIST developed by the WHO [23] and also validated in a digital version (Audio Computer-Assisted Self Interview [ACASI]) [24] which enables a thorough assessment for the service user of all psychoactive substance use. The system provides offline recruitment and online inclusion by health professionals, one-text messaging via a smartphone or by email, the completion of the self-assessment by clicking on the proposed link, sharing of the results with the health professional if the patient agrees, and the offer of a brief intervention or referral to an addiction center in case of identification of risk-prone usage.

The ASSIST enables 9 scores to be calculated corresponding to the different types of psychoactive substances (tobacco, alcohol, cannabis, cocaine, amphetamines, solvents, tranquilizers, hallucinogenic drugs, and opiates). A specific score for each substance was calculated by summing the scores for 6 questions. For example, scores of >4 (alcohol >10) reflect a moderate risk, and a high risk is indicated by scores >27 [23]. Depending on the risk profile, the participant is directed to a specific page. In case of low (or no) risk, a reassuring message inviting people to take the test again in a year's time is displayed. If the participant presents a moderate or high risk, they are encouraged to see the health professional again to discuss the results. To facilitate this, participants are given the opportunity to share their level of risk with the aforementioned health professional.

**Figure 1:**
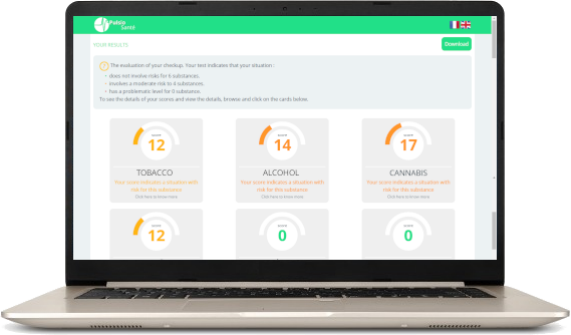
Screenshot of the Pulsio Santé application app.

After receiving results from a participant, the health professional should invite him or her to make an appointment (brief intervention or addiction center) within 2 weeks, provided that the assessment was determined to be some form of risk-related usage. To do this, the professional can use a digital guide on his or her Pulsio Santé app account and consult the participant’s responses to the self-assessment in detail, provided the person has agreed to share these responses (Figure 1).

The Pulsio Santé app has also been made commercially available to hospitals, occupational health institutions, and medical institutions to deploy prevention services for people at risk of addiction.

This study is the result of a tender response to a regional public health institution looking for subventions for operational and research projects dedicated to the prevention of addiction in several populations (employees, youth, low-income individuals) in real-life settings and through consultations. The study was therefore also aimed at demonstrating the soundness of the research to the public health institutions regarding the feasibility and efficacy of these types of novel digital health interventions (including but not limited to Pulsio Santé) deployed by private companies in real-life settings.

### Quantitative Study

Quantitative data were collected electronically. All actions, from the inclusion of participants, to the access to the screening test, to its completion, took place within the Pulsio Santé app. Therefore, use of the app, acceptance of the test (through connections to the app), and test results were all collected on the Pulsio Santé app.

Qualitative data from focus groups were collected and analyzed with the Sphinx iQ2 software.

#### Study Procedure

In September 2017, health professionals who volunteered to participate as investigators were invited to a meeting organized by the project team to receive information about the Pulsio Santé app and to standardize the manner in which the app was presented to participants.

An information and the consent form was added to the Pulsio Santé app. Service users could only be enrolled after having read the information and agreed to participate.

A case report form was created for each service user. All the information required by the protocol was gathered as encoded data. By signing this protocol, the principal investigator (JLV) and all the health professionals undertook to keep participant identities confidential.

As this self-assessment was initiated online by a text message or email request and outside the health professional’s consultation (but nevertheless linked to the consultation), the participants’ commitment to the procedure was ensured. It was an essential criterion for the participants’ access to the app. Similarly, any transmission of the results of the self-assessment to the health professional required the participants’ consent.

Users who opened the link were connected to a platform that allowed them to answer the ASSIST in its digital version (ACASI) in less than 5 minutes. If participants then agreed, they could transfer their results to their health professional. If the service users had not initially agreed to this, messages appeared allowing them to do so after completion of their self-evaluation or would invite them to get in touch with their general practitioner should the assessment detect a risk.

#### Evaluation Criteria

##### Acceptability of the Pulsio Santé Web-Based App

Two levels of appreciation of the acceptability were assessed. They concerned the health professionals’ ability to propose the app to service users they encounter in their daily practice and the users’ ability to use it. The percentage of salaried workers and students who agreed to receive a text message (level 1) and the percentage of those who clicked on the proposed link (level 2) were thus collected.

##### Feasibility of the Pulsio Santé Web-Based App

Two levels of appreciation of the feasibility of the Pulsio Santé app were explored. They concerned the ability of the app to enable those to whom the app was offered to carry out a complete assessment of their psychoactive substance use and to share their results with the health professional who had offered it to them. The percentage of salaried workers and students who filled in the ASSIST completely (level 1) and the percentage of users who shared their results with their health professional (level 2) were thus collected.

Furthermore, to meet the secondary objective, the percentage of service users who agreed to a brief intervention was calculated in cases of risk-related status, and the percentage of those presenting problematic substance use who agreed to be referred to an addiction center was also calculated.

#### Data Collection

The data were collected by health professionals from the SSTRN and SUMPPS involved as investigators during their day-to-day work with workers and students as well as by service users themselves through completing their screening autonomously.

All data were collected already encoded and stored in data servers (European HIPAA [Health Insurance Portability and Accountability Act]-like regulations) located in France.

#### Statistical Analysis

Statistical analyses used the usual techniques of descriptive statistics (frequency, mean, and SD). The ASSIST-specific substance scores were computed according to the scoring method [23]. A *t* test was performed to anonymously compare substance scores, and a chi-square test was used to compare percentages across the groups of sharers and nonsharers. The statistical significance was set at α =.05. Statistical analyses were performed with R version 3.4.3 (The R Foundation for Statistical Computing).

### Qualitative Study

#### Data Collection

A panel of health professionals participated in semistructured focus groups (or 1 interview) on the acceptability and feasibility of the web-based app between February 2018 and March 2018. A first focus group with the SUMPPS professionals was organized with 5 nurses. The participation rate was 83.3% (5/6). In the SSTRN department, a focus group was organized with 7 SSTRN professionals (4 nurses and 3 physicians), and 1 face-to-face interview with a nurse. The participation rate of the SSTRN professionals was 22.0% (8/36). Focus group topics covered the following: usefulness and advantages of the app; problems, limitations, and frustrations; necessary improvements to the app; and integration into routine practice. The content was recorded and transcribed for analysis. After each focus group, the interviewers checked the transcripts for quality and clarity.

#### Qualitative Analysis

A multicategory content analysis of the focus group corpus using a frequency distribution table method was conducted to form an exhaustive corpus of what had been said and to identify the main themes and subthemes around which the representations of professionals and participants were organized. A theme frequency analysis was conducted, and possible links between the categories were sought. Results were compared by 2 researchers (EA and ML) to ensure intercoder validity.

Qualitative analyses were performed on Sphinx iQ2 survey and data analysis software.

### Ethics Review

All study procedures fully complied with the requirements of French law. The relevant authority for this research was the National Data Protection Committee (Commission Nationale de l'Informatique et des Libertés [CNIL]). The CNIL declaration of conformity of the Study was registered under reference number 2094055 dated August, 28, 2017. No ethics Approval or registration of clinical trials was necessary for this research, as it is not within the scope of application of the French Public Health Code.

## Results

### Characteristics of the Sample

A total of 1474 service users who came to see either a health professional in the SSTRN department (n=1067) or a nurse in the SUMPPS department (n=407) were asked to participate.

Forty-two health professionals participated as investigators in the study. Among them, 14% (6/42) were working in the SUMPPS and 86% (36/42) in the SSTRN.

### Acceptability of the Pulsio Santé Web-Based App

**Figure 2:**
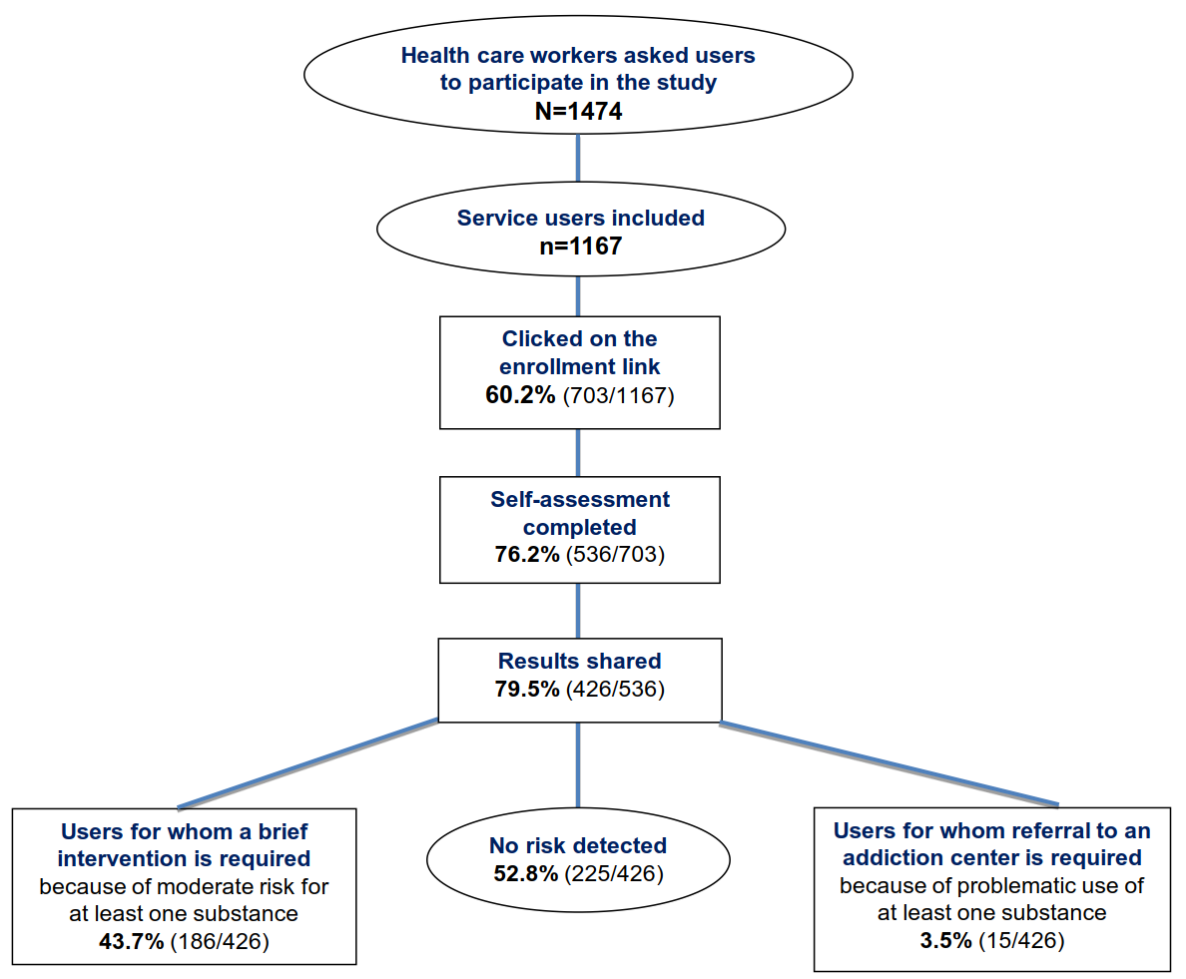
Flowchart of the acceptability and feasibility study - 20210928.

The percentage of participants who agreed to receive a text message or an email, considered as the first level of acceptability, was 79.17% (1167/1474; Figure 2). The participation rates for salaried workers and students were 76.85% (820/1067) and 85.2% (347/407), respectively. The sample comprised 70.26% (820/1167) salaried workers and 29.73% (347/1167) students.

The percentage of participants who clicked on the subscriber link, considered as the second level of acceptability, was 60.24% (703/1167; Figure 2). The participation rates for salaried workers and students were 54.0% (443/820) and 74.9% (260/347), respectively. The sample comprised 63.0% (443/703) salaried workers and 37.0% (260/703) students.

### Feasibility of the Pulsio Santé Web-Based App

The percentage of participants who completed their self-evaluation entirely, considered as the first level of feasibility, was 76.2% (536/703; Figure 2). The participation rates for salaried workers and students were 73.8% (327/443) and 80.4% (209/260), respectively. The sample comprised 61.0% (327/536) salaried workers and 39.0% (209/536) students.

The percentage of participants who shared the results of their evaluation with their health professional, considered as the second level of feasibility, was 79.5% (426/536; Figure 2). The participation rates for salaried workers and students were 80.1% (262/327) and 78.5% (164/209), respectively. The sample comprised 61.5% (262/426) salaried workers and 38.5% (164/426) students.

Students clicked on the registration link significantly more often (260/347, 74.9%) than did salaried workers (443/820, 54%; *P*<.001), but there was no statistical difference between students and salaried workers for the number of completed self-evaluations (*P*=.06) or the number of shared self-evaluation results (*P*=.72). For all substances except tobacco (*P*=.02), the students’ scores were not significantly different from those of the salaried workers' scores.

### Health Professionals’ Perceptions of the Feasibility of the Web-Based App

Focus groups with the health professionals explored 4 themes to evaluate their points of view on the acceptability and feasibility of the Pulsio Santé app.

First, the usefulness and the advantages of the app were studied. It appeared that the app was easy and quick to propose and that participants were easy to enroll in the study (see quotation 1, Table 1).

**Table 1:** Quotations illustrating the professionals’ perceptions about the acceptability and the feasibility of the app.

Quotation number		English translation with original French quotation below
**1**		
	English	Including patients was kind of easy; it didn’t really take long.
	French	Les inclusions, c’était assez facile, ça ne prenait vraiment pas longtemps.
**2**		
	English	It added a little extra to the individual consultation with the patient by opening up the exchange a little.
	French	Ça a apporté un plus au niveau de la consultation du rendez-vous individuel, en ouvrant un petit peu plus l’échange.
**3**		
	English	I’d say it’s rather beneficial to patients themselves; self-assessment is interesting on a personal level; it helps to know where we are.
	French	J’aurais plutôt dit l’avantage pour la personne elle-même, l’autotest aussi qui est intéressant, pour soi, de savoir où on en est.
**4**		
	English	It also encourages people to think.
	French	Ça amène les gens à réfléchir aussi.
**5**		
	English	I wonder whether we shouldn't offer the survey before consultations… well in advance.
	French	Moi je me pose la question de savoir si on ne devrait pas proposer l’enquête avant les rendez-vous en fait.
**6**		
	English	It was the same for me. I didn’t get any response to the appointments I suggested.
	French	Moi c’est pareil, je n’ai pas eu de réponses au rendez-vous proposé.
**7**		
	English	We can do a bit of digging around the addiction problems, but then, what do we do next? If the person does not come back for a brief intervention… question mark. Screening, why not? But then, what means do we have as health professionals?
	French	On va aller creuser un petit peu sur les addictions et puis après, qu’est-ce qu’on en fait ? Si la personne ne revient pas en intervention brève, point d’interrogation. Faire du dépistage, ok, mais après, qu’est-ce qu’on a, nous, comme moyens ?
**8**		
	English	Even if people don’t share their result, it can still be interpreted. I mean, used it even so. But to do this, we need to know if the person enrolled and gave his answers or enrolled and did nothing. That's important. Honestly, I was surprised; we couldn’t see if he had answered.
	French	Même sans partager, ça peut être interprété, enfin utilisé quand même. Mais pour ça, il faut qu’on voie s’il s’est inscrit et s’il a répondu, ou s’il s’est inscrit et qu’il n’a rien fait. Ça c’est important. Franchement, moi ça m’a étonnée quand même, on ne voit pas s’il a répondu.
**9**		
	English	He scored as “at risk for tobacco use” despite the fact that he also uses speed, cannabis, and cocaine, which worries me far more, but these did not score as risky. There is definitely something wrong that needs to be looked at.
	French	Il m’est apparu comme « situation à risque pour le tabac », alors qu’il consomme des amphétamines, du cannabis, de la cocaïne, qui m’interpellent largement plus et qui, là, indique qu’il n’y a pas de risque. Il y a quelque chose à revoir vraiment là-dessus.
**10**		
	English	I think it would be good for these people to be given links to prevention websites at the end of the questionnaire or a list of institutions that might help. Given that we are including people via a web-based app, it would be good to have links that enable them to know which institutions specialize in treating addictions.
	French	Je pense qu’à la fin du questionnaire, ce serait bien qu’il y ait des liens qui leur permettent d’accéder à des sites de sensibilisation, à des organismes qui peuvent venir en aide. Dans la mesure où l’on est dans une transmission par voie numérique, c’est effectivement de mettre des liens qui permettraient d’avoir connaissance des organismes d’addictologie existant.
**11**		
	English	I would be willing to use this tool in my daily practice, providing adaptations are made to our area of work, adapted to occupational medicine.
	French	Je serais prête à utiliser cet outil au quotidien, avec une adaptation par rapport à notre métier, adapté santé au travail.
**12**		
	English	I experienced some difficulties introducing the tool, when to mention it… So I tried different approaches.
	French	J’avais des difficultés pour présenter l’outil, à quel moment dire... Donc j’ai un peu tâtonné.

The app facilitated discussion on the use of substances, complemented the medical consultation (see quotation 2, Table 1), and made participants think about their substance use (see quotation 3 and 4, Table 1).

Second, problems, limitations, and frustrations were discussed. Self-evaluation after the medical consultation was not considered as an optimal strategy (see quotation 5, Table 1). The messages (mail, email, or phone calls) sent by the health professionals to users with moderate or high risk were not followed up (see quotation 6, Table 1). Health professionals also frequently wondered what to do with a positive result from a participant (see quotation 7, Table 1).

Health professionals were then interviewed on the necessary improvements of the app. They suggested making a distinction between nonresponders and nonsharers (see quotation 8, Table 1). This is impossible, ethically speaking, if the patient refuses to share his or her results.

A discrepancy between the results on the WHO measure and actual use by participants was highlighted (see quotation 9, Table 1). At the end of the self-evaluation, several professionals suggested that links to government preventive medicine and public health websites should be created (see quotation 10, Table 1).

Finally, the integration into routine practice was debated. Focus group participants pointed out that the app should be customized according to the medical department concerned (see quotation 11, Table 1) and that professionals should be trained before offering the app to their service users (see quotation 12, Table 1).

### Health Professionals’ Practice

The other secondary objective focused on the way the Pulsio Santé app could facilitate access to a brief intervention or referral to an addiction center when these are recommended on the basis of the evaluation results. Health professionals offered 51 brief intervention appointments to 186 substance users identified as being at moderate risk for at least 1 substance (51/186, 27.4%). A total of 5.4% (10/186) of the brief interventions considered necessary were carried out by the health professionals, half of which were mediated via the Pulsio Santé app. Health professionals offered 2 addiction center appointments to 15 substance users identified as having problematic use of at least 1 substance (2/15,13.3%). The 2 proposed appointments were accepted by the users, and all were mediated by the Pulsio Santé app. More descriptive results are available in Multimedia Appendix 1.

## Discussion

### Principal Results

This acceptability and feasibility study, carried out on a large size sample across 2 institutions with different service user profiles, demonstrated high acceptability of the Pulsio Santé web-based app by health professionals, as well as by the salaried workers and students to whom it was offered. In terms of feasibility, the data were similar across workers and students: once the service users had clicked on the enrolment link, a high participation rate in the self-assessment of (536/703, 76%) and a high rate of results shared with the health professional (426/536. 79.5%) were observed. The fact that this applied to both students and occupational medicine participants, where reluctance to confide for fear of their employer being informed is often observed, is also worth noting. It is also important to note that the service users who filled in their self-assessment completely without sharing their results with their health professionals were not very different from those who did share, except for those who indicated tobacco use. It is finally worth noting that the percentage of service users in a problematic situation who did not wish to share their results is not significantly different from those who did, which reinforces the relevance of the app.

The fact that this assessment can be performed in a few minutes as an online self-assessment, with only 1 text message needed to access the screening test and outside consultation time with the health professional, guarantees that it will be acceptable to users. The presence of the health professional offers the possibility for help or care if necessary.

The ASSIST and its digital version (ACASI) enable a quick and precise assessment of the use of all the main psychoactive substances (including medications that are misused).

### Limitations

There were no missing values in the screening questionnaire, as service users had to answer all the questions for the psychoactive substances they had checked at the first question. They could not go to the following steps and, therefore, to the final results if they did not answer all their questions. This can be interpreted as a limitation given that even partial results and scores on certain substances might be sufficient to trigger the willingness to get some help. We could imagine that some people bored with the whole questionnaire or misunderstanding parts of it would like to submit their partial answers to get partial results (and act on them). In our study, their only choice in this situation was to drop out completely or persist to obtain the full results.

Furthermore, while early detection is clearly facilitated by this app, there are nevertheless many unanswered questions. First, inclusion refusals, although not particularly numerous, were to be documented by health professionals, which was not in fact done. Second, the risk thresholds that were defined a decade ago for the ASSIST raise issues of relevance. Indeed, nearly half of the service users were at moderate risk with at least 1 substance, and some substance users who had a score above 20 for at least 3 psychoactive substances did not appear as “problematic”. Finally, the proposals (brief intervention or addiction center) justified by the results of the assessment were very often not implemented. Although this was not the main objective of the study, it is nevertheless a crucial issue for the impact of early detection in public health prevention.

Among the improvements suggested during the focus groups, the possibility of offering the app before rather than during the meeting with a health professional was mentioned by several professionals. This would allow for the implications of the assessment to be quickly taken into account. Besides the purely practical and ethical questions raised by this possibility (such as collective answers by groups of students in the waiting room), studies on other populations should be carried out. To this end, the Pulsio Santé app has been deployed in larger populations (such as rural areas) and will be evaluated in those new settings.

Moreover, defining the stage when the screening test should be introduced during follow-up and adapting the web-based app to the aims of each department and to their service users were among the strong expectations that were expressed during the focus groups. These two points indeed seem essential to guaranteeing efficient coordination between detection and care when the latter is indicated.

### Conclusions

This study on a large-size sample showed good acceptability and feasibility of the Pulsio Santé web-based app by service users and health professionals.

The qualitative study, however, showed that there were obstacles on the professionals’ side, which could justify the need for further studies more directly focused on these limitations and the web-based app as a mediator-facilitator; studies on other populations should be carried out and designed to extend to purely behavioral addictions (ie, with no psychoactive substance use).

## Appendix

Multimedia Appendix 1 Quantitative results from the screening test.

## Abbreviations

ACASI: Audio Computer-Assisted Self Interview
ASSIST: Alcohol, Smoking and Substance Involvement Screening Test
CNIL: Commission Nationale de l'Informatique et des Libertés
HIPAA: Health Insurance Portability and Accountability Act
SSRTRN: Service de Santé au Travail de la Région Nantaise
SUMPPS: Service de Santé des Étudiants
WHO: World Health Organization
