# Supplementary material for: Assessing the Acceptability and Feasibility of a Web-Based Screening for Psychoactive Substance Users Among a French Sample of University Students and Workers: Mixed Methods Prospective Study
Source: JMIR Form Res. 2021 Oct 1;5(10):e15519. doi: 10.2196/15519 (PMC8520132; doi:10.2196/15519)
Supplement: Multimedia Appendix 1 [file formative_v5i10e15519_app1.docx]

Appendix – Quantitative results from the screening test

All participants

Among all participants who answered the entire screening test, scores on each substance recorded in the ASSIST were as follow:

|  | Min | Median | Average | Max | SD |
| --- | --- | --- | --- | --- | --- |
| **Substances** | | | | | |
| Tobacco | 0 | 2 | 6.3 | 31 | 8.3 |
| Alcohol | 0 | 4 | 5.8 | 35 | 5.6 |
| Cannabis | 0 | 0 | 1.9 | 35 | 5.1 |
| Cocaine | 0 | 0 | 0.3 | 31 | 2.2 |
| Amphetamine type stimulants | 0 | 0 | 0.2 | 26 | 1.5 |
| Inhalants | 0 | 0 | 0 | 5 | 0.3 |
| Sedative pills | 0 | 0 | 0.4 | 21 | 1.9 |
| Hallucinogen | 0 | 0 | 0.1 | 6 | 0.4 |
| Opioids | 0 | 0 | 0.2 | 24 | 1.5 |
| Other | 0 | 0 | 0.1 | 21 | 1.4 |

Table 2 – Scores on each substance

|  | Yes | No | Total |
| --- | --- | --- | --- |
| Participants with a moderate risk on at least one substance, **n (%)** | 252 (47) | 284 (53) | 536 (100) |
| Participants with a high risk on at least one substance, **n (%)** | 18 (3.4) | 518 (96.6) | 536 (100) |

Table 3 – Different levels of risks

|  | 1 | 2 | 3 | 4 | 5 | 6 | Total |
| --- | --- | --- | --- | --- | --- | --- | --- |
| Participants with a moderate risk by number of substances | 158 | 61 | 25 | 6 | 1 | 1 | 252 |
| % of participants with a moderate risk by number of substances | 62.7 | 24.2 | 9.9 | 2.4 | 0.4 | 0.4 | 100 |
| Participants with a high risk by number of substances | 16 | 2 | 0 | 0 | 0 | 0 | 18 |
| % of participants with a high risk by number of substances | 88.9 | 11.1 | 0 | 0 | 0 | 0 | 100 |
| Participants (total) with a moderate or high risk by number of substances | 158 | 62 | 27 | 7 | 3 | 1 | 256 |
| % of participants (total) with a moderate or high risk by number of substances | 60.9 | 24.2 | 10.5 | 2.7 | 1.2 | 0.4 | 99.9 |

Table 4 – Amount of participants segmented by the number of substances at a moderate and high risk

Among all participants who answered the entire screening test, they could either decide to share their results/risks or not with the health profesionnal that included them :

|  | Number | Percentage |
| --- | --- | --- |
| Did not accept to share his risks/results | 110 | 20.5 |
| Accepted to share his risks/results | 426 | 79.5 |

Participants who did not share their risk/results

Descriptive analysis of participants’ data who did not accept to share their risk/result with their health professional

110 participants who did not share their risk/result participated to the study.

|  | Min | Median | Average | Max | SD |
| --- | --- | --- | --- | --- | --- |
| Tobacco | 0 | 6 | 8.3 | 27 | 8.6 |
| Alcohol | 0 | 4 | 6.4 | 27 | 5.6 |
| Cannabis | 0 | 0 | 3.2 | 32 | 6.4 |
| Cocaine | 0 | 0 | 0.2 | 17 | 1.6 |
| Amphetamine type stimulants | 0 | 0 | 0.3 | 12 | 1.5 |
| Inhalants | 0 | 0 | 0.1 | 3 | 0.4 |
| Sedative pills | 0 | 0 | 0.6 | 21 | 2.4 |
| Hallucinogen | 0 | 0 | 0 | 3 | 0.3 |
| Opioids | 0 | 0 | 0.3 | 24 | 2.3 |
| Other | 0 | 0 | 0 | 2 | 0.2 |

Table – 5 Scores on each substance

|  | Yes | | No | | Total | |
| --- | --- | --- | --- | --- | --- | --- |
|  | n % | | n % | | n % | |
| Participants with a moderate risk on at least one substance | 66 | 60 | 44 | 40 | 110 | 100 |
| Participants with a high risk on at least one substance | 3 | 2.7 | 107 | 97.3 | 110 | 100 |

Table 6 – Different levels of risks

|  | 1 | 2 | 3 | 4 | 5 | 6 | Total |
| --- | --- | --- | --- | --- | --- | --- | --- |
| Participants with a moderate risk by number of substances | 40 | 18 | 4 | 4 | 0 | 0 | 66 |
| % of participants with a moderate risk by number of substances | 60.6 | 27.3 | 6.1 | 6.1 | 0 | 0 | 100.1 |
| Participants with a high risk by number of substances | 2 | 1 | 0 | 0 | 0 | 0 | 3 |
| % of participants with a high risk by number of substances | 66.7 | 33.3 | 0 | 0 | 0 | 0 | 100 |
| Participants (total) with a moderate or high risk by number of substances | 40 | 16 | 5 | 4 | 1 | 0 | 66 |
| % of participants (total) with a moderate or high risk by number of substances | 60.6 | 24.2 | 7.6 | 6.1 | 1.5 | 0 | 100 |

Table 6 – Amount of participants segmented by the number of substances at a moderate and high risk

Participants who shared their risk/results

Descriptive analysis of participants’ data who accepted to share their risk/result with their health professional

426 participants shared their risk/result and participated to the study.

|  | Min | Median | Average | Max | SD |
| --- | --- | --- | --- | --- | --- |
| Tobacco | 0 | 0 | 5.8 | 31 | 8.2 |
| Alcohol | 0 | 4 | 5.6 | 35 | 5.6 |
| Cannabis | 0 | 0 | 1.5 | 35 | 4.6 |
| Cocaine | 0 | 0 | 0.3 | 31 | 2.4 |
| Amphetamine type stimulants | 0 | 0 | 0.2 | 26 | 1.4 |
| Inhalants | 0 | 0 | 0 | 5 | 0.3 |
| Sedative pills | 0 | 0 | 0.4 | 15 | 1.8 |
| Hallucinogen | 0 | 0 | 0.1 | 6 | 0.5 |
| Opioids | 0 | 0 | 0.2 | 15 | 1.3 |
| Other | 0 | 0 | 0.1 | 21 | 1.5 |

Table 7 – Scores on each substance

|  | Yes | | No | | Total | |
| --- | --- | --- | --- | --- | --- | --- |
|  | n % | | n % | | n % | |
| Participants with a moderate risk on at least one substance | 186 | 43.7 | 240 | 56.3 | 426 | 100 |
| Participants with a high risk on at least one substance | 15 | 3.5 | 411 | 96.5 | 426 | 100 |

Table 8 – Different levels of risks

|  | 1 | 2 | 3 | 4 | 5 | 6 | Total |
| --- | --- | --- | --- | --- | --- | --- | --- |
| Participants with a moderate risk by number of substances | 118 | 43 | 21 | 2 | 1 | 1 | 186 |
| % of participants with a moderate risk by number of substances | 63.4 | 23.1 | 11.3 | 1.1 | 0.5 | 0.5 | 99.9 |
| Participants with a high risk by number of substances | 14 | 1 | 0 | 0 | 0 | 0 | 15 |
| % of participants with a high risk by number of substances | 93.3 | 6.7 | 0 | 0 | 0 | 0 | 100 |
| Participants (total) with a moderate or high risk by number of substances | 116 | 46 | 22 | 3 | 2 | 1 | 190 |
| % of participants (total) with a moderate or high risk by number of substances | 61.1 | 24.2 | 11.6 | 1.6 | 1.1 | 0.5 | 100.1 |

Table 9 – Amount of participants segmented by the number of substances at a moderate and high risk
